# Improving Aeroelastic Stability of Bladed Disks with Topologically Optimized Piezoelectric Materials and Intentionally Mistuned Shunt Capacitance

**DOI:** 10.3390/ma15041309

**Published:** 2022-02-10

**Authors:** Xin Liu, Yu Fan, Lin Li, Xiaoping Yu

**Affiliations:** 1 School of Energy and Power Engineering, Beihang University, Beijing 100191, China; liu_xin_flying@buaa.edu.cn (X.L.); feililin@buaa.edu.cn (L.L.); 2 Beijing Key Laboratory of Aero-Engine Structure and Strength, Beijing 100191, China; 3 China Academy of Aerospace Aerodynamics, Beijing 100074, China; xiaopingyu@buaa.edu.cn

**Keywords:** aeroelastic stability, bladed disk, intentional mistuning, piezoelectric material, topological optimization

## Abstract

It is well known that bladed disks with certain patterns of mistuning can have higher aeroelastic stability than their tuned counterparts. This requires small but accurate deviation of the mechanical properties on each blade sector, and currently it is difficult to realize by mechanical manufacturing. In this paper, we propose an adaptive strategy to realize the intentional mistuning for the improvement of aeroelastic stability. The basic idea is to bond or embed piezoelectric materials to each blade and use different shunt capacitance on each blade as the source of mistuning. When the shunt capacitance varies from zero (open-circuit, OC) to infinity (short-circuit, SC), the stiffness of each blade changes within a relatively small interval. In this way, the required small difference of stiffness among blades is altered into a relatively larger difference of the shunt capacitance. This provides a more feasible and robust way to implement the intentional mistuning, provided that the variation interval of blade stiffness between OC and SC contains the limits of required mistuning. Thus, it is critical to maximize the ability of changing the blade stiffness by shunt capacitance with limited amount of piezoelectric materials. To do so, a straightforward approach is proposed to get the best distribution of piezoelectric materials on the blade for the targeting mode. This approach is based on the FE model of the bladed disc, and the piezoelectric materials are introduced by replacing elements (if they are embedded) or adding an extra layer of elements (if they are bonded). An empirical balded disc with NASA-ROTOR37 profile is used as the example. With a proper design of the mistuning pattern and replace use piezoelectric materials of only 10% the blade mass, the proposed method can significantly improve the aeroelastic stability of bladed disks.

## 1. Introduction

Improving aero-elastic stability to avoid flutter is an essential task for bladed disks in modern high-performance aero-engines. Previous studies [1,2] have proved the beneficial effects of mistuning on aeroelastic stability of cascades. It was frequently reported that mistuning can increase the minimum aerodynamic damping of the cascades while decreasing the maximum one [3,4]. Mistuning refers to slight mechanical difference among blade sectors, and it is unavoidable due to manufacturing tolerances and in-service wear [5,6]. Such intrinsic mistuning is randomly distributed and thus uncontrollable. As the frequency migration caused by mistuning is identified as the crucial factor influencing the aeroelastic stability [7,8], researchers have to intentionally impose certain mistuning pattern to maximum frequency migrations so as to achieve the best beneficial effects. Researchers have studied alternate patterns [9,10,11,12,13], sinusoidal patterns [14,15] and others [16].

Extensive experimental evidences have been reported regarding this topic, and the implementation of mistuning pattern is among the key techniques. Groth et al. [11] milled grooves the shroud to realize the intentional mistuning. Their results show that a well-designed mistuning can push the unstable boundary away from the turbine operating envelope. Figaschewsky et al. [17] applied heavy paint to the blades to realize the intentional mistuning. This is a non-destructive approach, but the passage of fluid may be narrowed. Adding mass to the tips of blades is also adopted to realize the intentional mistuning by some researchers [12,18]. Note that intentional mistuning requires small but accurate deviation of the mechanical properties on each blade sector. It can be a difficult task for mechanical manufacturing because it may prone to additional errors for small amount of manufacturing. Moreover, the best mistuning pattern may vary for different vibration modes, or at different working conditions. Pure mechanical implementation lacks the capability to adjust.

With the two-way energy transfer capability between the mechanical and electric fields, piezoelectric materials can be used as an adaptive way to tailor the mechanical properties by shunting different external circuits. For example, connecting a resistor to the electrodes is equivalent to adding an external viscous damper to the host structure. Likewise, a resistor-inductor circuit is equivalent to an external oscillator [19]. Finally, a capacitor is equivalent to an external grounded spring, thus changing the capacitance can adjust the natural frequencies of the host structure.

On this basis, we propose an alternative and more robust approach to implement mistuning. The basic idea is to bond or embed piezoelectric materials to each blade, and use different shunt capacitance on each blade as the source of mistuning. When the shunt capacitance varies from zero (open-circuit, OC) to infinity (short-circuit, SC), the stiffness of each blade changes within a relatively small interval. In this way, the required small difference of stiffness among blades is altered into a relatively larger difference of the shunt capacitance. This provides a more feasible and robust way to implement the intentional mistuning, provided that the variation interval of blade stiffness between OC and SC contains the limits of required mistuning.

The change in mechanical properties is limited by the model electromechanical coupling factor (MEMCF), which quantifies the energy exchange capability of a given piezoelectric structure. MEMCF is defined as the fraction of eigenvalue (square of natural frequencies) deviation from OC to SC. It can also be demonstrated that an external (positive) capacitance can only changes a modal frequency of the host structure from the values with OC to it with SC. Thus, maximizing the ability of changing the blade natural frequencies by shunt capacitance is equivalent to maximizing the MEMCF. This is critical especially when limited amount of piezoelectric materials are allowed to use in future engineering practice.

Existing studies [20,21,22] have pointed out that MEMCF (for a given mode) only depends on the number, shape, size, and location of the attached piezoelectric materials. Therefore, maximizing MEMCF is leading us to designing the geometrics of piezoelectric materials, as reported in the applications of modal sensing [23], actuating [24], energy harvesting [25], vibration mitigation [26,27,28,29], and so on. In these studies, the host structures to place piezoelectric materials are relatively simple and the researchers mainly takes position and direction of the given shape piezoelectric patches. For more complex structures like bladed disk, more factors such usage, shape, connected ways, arrangement method, etc., should be considered in the optimization process. Moreover, these methods are based on certain optimization processes such as the genetic algorithms, therefore modal analysis will be repeatedly conducted with updated design variables. Despite that modal analysis is much faster than forced response, for complex structures like bladed disks (the FE model can have millions of DOFs), it is still a heavy task.

In this work, an alternative approach is proposed and it only requires a single modal analysis in prior. First, we point out that MEMCF is also the fraction of electric energy over elastic potential energy associate with the structural mode. Consequently, piezoelectric materials should be placed in priority to the places with higher modal electric energy to achieve highest possible MEMCF. In this way, the shape of piezoelectric material is determined only by a single modal analysis, and we do not need to invoke any standard topological optimization algorithm. Iteration is avoided and the proposed approach has added very little computing load. This can be the major advantage of the proposed approach.

The claimed originality of the work is two-fold. The first one is using capacitance variation of piezoelectric shunt to modify the mechanical properties of each blade. To the authors knowledge, there is no other open literature exploring this idea than a preliminary work done by the authors [30]. This former publication has three shortages: (1) no unstable modes, namely, a stable working condition of the fluid field is selected; (2) the shape of piezoelectric materials is not optimized; and (3) capacitance is directly following the harmonic mistuning pattern rather than being determined by the harmonic mistuning pattern of mechanical properties. To further demonstrate the feasibility of such an original idea with more convincing data, we conduct the work reported in this manuscript and resolved all three aforementioned shortages. In particular, the topological design approach with just a single modal analysis can be attributed to the second original contribution of the conducted work. As mentioned in the previous review and the following detailed presentation, this design approach is significantly different from the existing method in current literature and it is especially suitable for the problem raised in this manuscript.

In later parts of this paper, an empirical bladed disk with NASA-ROTOR37 profile (Section 1) is introduced, and it is used as an example to illustrate and validate the proposed approach. The theory and procedure of the topological optimization approach are enclosed in Section 3.1 and Section 3.2. Bladed disks with optimized distribution of piezoelectric materials are given at Section 3.3. The performance of intentional mistuning realized by the mistuning of external capacitance is presented in Section 3.1 and Section 3.2. Eventually, the robust of this adaptive method in the presence of random mistuning is also examined (Section 3.3).

## 2. Problem Formulation

**Figure 1:**
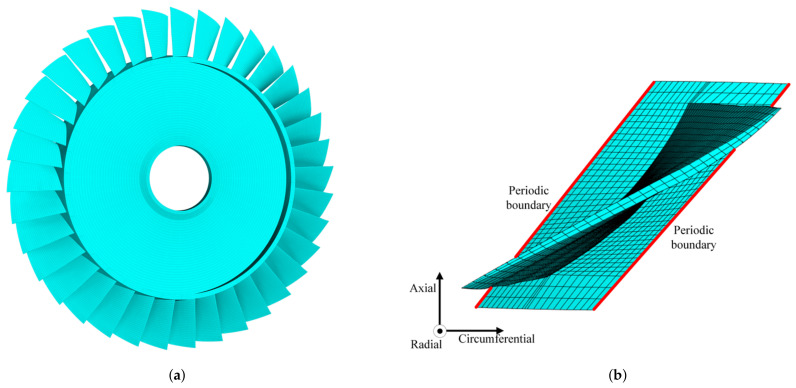
The FEM model of the Rotor37 bladed disk. (**a**) Overall model. (**b**) Sector model.

The bladed disk shown in Figure 1 is considered in this work. It consists of 36 blades with NASA-Rotor37 profile and a dummy disk. The material parameters of the bladed disk are as follows: modulus of elasticity $2.8\times 10^{5}\;\mathrm{MPa}$, density $7.8\times 10^{-9}\;{t/\mathrm{mm}}^{3}$ and Poisson ratio 0.3. In the following analysis, the displacement at inner diameter of disk is constrained, in order to simulate the actual installation conditions.

**Figure 2:**
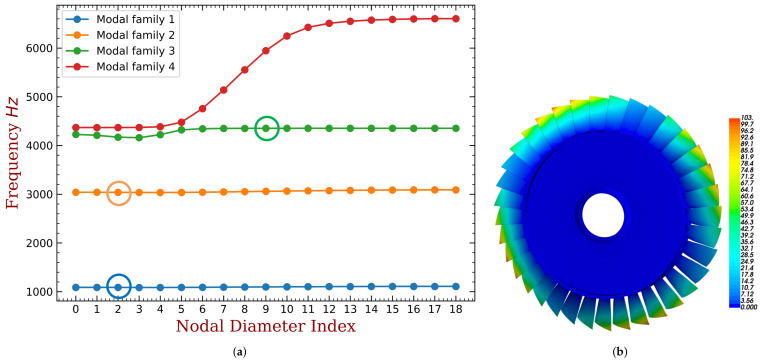
Modal analysis results of the empirical bladed disk. (**a**) The frequency versus nodal diameter index. (**b**) Modal shape marked with blue circle.

First, we conduct modal analysis of the tuned bladed disk with no fluid–structure interaction, as shown in Figure 2a. We have checked the mesh density and a finer mesh can only provide very minor improvements of the results. The modes with similar blade deformation but different engine-order (the number of nodal lines when the bladed disk vibrates) are classified to the same modal family, and they are link by the same line in Figure 2a. In this work, we use the aeroelastic stability of the first modal family to illustrate the proposed approaches. The modes in this family have similar frequency for they are all dominated by the blade bending deformation as shown in Figure 2b.

Second, we access the aeroelastic stability of the tuned bladed disk as a reference. To do this, aerodynamic influence coefficient (AIC) [31] is employed to model the aeroelastic force caused by the movement of blades, leading to a linearized dynamic equation of the bladed disk for free vibration:

(1) $$\hat{M}\ddot{y}+\left(\hat{K}+L\right)y=0$$

where $\hat{M}$ and $\hat{K}$ are mass and stiffness matrices of the bladed disk; y is the displacement vector. Matrix L is constructed by the AICs and it contains complex numbers. Analyzing the modal characteristics in this case will lead to a complex eigenvalue problem:

(2) $$(-{\hat{\omega }}_{j}^{2}\hat{M}+\hat{K}+L)y=0$$

and the natural frequencies ${\hat{\omega }}_{j}$ may become complex values. Aerodynamic damping ratio $\xi_{j}$ of the *j*th mode can be obtained by

(3) ξj=−Im(ω^j)|ω^j|

The system become unstable if there are negative values of aerodynamic damping ratio. Therefore, we will use the minimum value of aerodynamic damping ratio among all the modes, denoted by $\xi_{\min}$, as an indicator for the aeroelastic stability of the system. If $\xi_{\min}<0$ then the system is unstable, and if $\xi_{\min}$ increases after some treatment we can conclude that the aeroelastic stability is improved.

**Figure 3:**
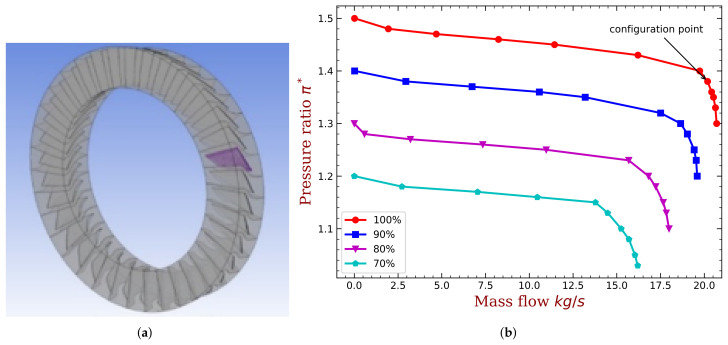
Outline of the CFD analysis of the bladed disk. (**a**) Flow field calculation domain. (**b**) The performance curves.

This method is relatively mature and details can be found in the literature [32]. For the sake of brevity, we do not repeat the basics of this method but only presents the key results. Specifically, a numerical simulation of the flow field should be carried out to determine AICs, and the calculation domain is shown in Figure 3a. The performance characteristics of the bladed disk are analyzed and shown in Figure 3b. The lines refer to different operational rotation speed, and the speed is proportional to the design speed of the rotor. When the bladed disk operates under specific rotation speed, the pressure ratio would drastically decrease as the mass flow increase to the block margin. In addition, the pressure ratio would keep almost constant as the mass flow decreases to the unstable region. When the rotor operates close to the unstable region, the bladed disk is more prone to flutter.

**Figure 4:**
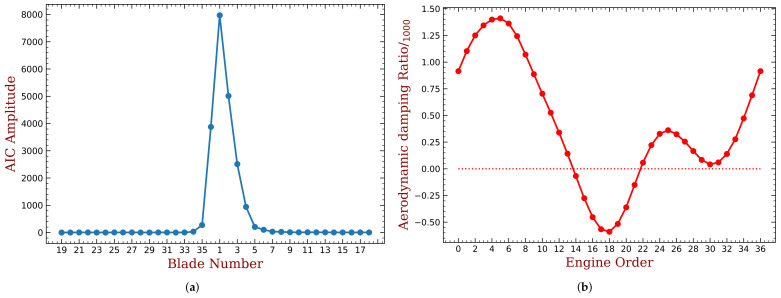
The aeroelastic stability analysis of the tuned bladed disk. (**a**) Aerodynamic influence coefficients. (**b**) Aerodynamic damping.

We choose the case (marked in Figure 3b) close to the unstable region as the working point to illustrate the feasibility of the adaptive method in improving the aeroelastic stability of the bladed disk. The boundary conditions and more details about the flow field settings can be found in our previous publication [33]. The aerodynamic influence coefficients for each blades when only the 1st blade is vibrating with the first bending mode are shown in Figure 4a. Thus, each AIC represents the aeroelastic force acted on each blade, generated by the movement of the 1st blade, and projected to the same modal coordinate (the first bending mode). The AICs presented in Figure 4a can be regarded as a row of matrix L in Equation (2). Changing between different moving blades can yield other rows of matrix L. Due to cyclic symmetry, all the remaining rows of matrix L can be generated by shifting the order of AICs presented in Figure 4a. The blade with larger distance to the reference (1st) blade has smaller aerodynamic force, and this is expected. Because of the blade torsion, the AIC values are not symmetric with respect to blade index 1, namely, AIC of blade 2 does not equal to blade 36. This means that L is not a symmetric matrix and the eigenvalues of Equation (2) are no longer double roots. The aerodynamic damping is computed and shown in Figure 4b. In this working condition, it is notable that there exists unstable modes with negative the aeroelastic damping. In our later investigation, we will use the proposed method to alleviate the unstable modes.

## 3. Topology Optimization for the Piezoelectric Materials

### 3.1. Theoretical Basis

A structure with piezoelectric materials has two coupled physical fields: the mechanical field and the electric field. The coupling strength between these two fields for the jth mode is quantify by modal electromechanical coupling factors (MEMCF), and it is defined as [22,34,35,36]

(4) $$k_{j}^{2}=\frac{{\left(\omega_{j}^{\mathrm{OC}}\right)}^{2}-{\left(\omega_{j}^{\mathrm{SC}}\right)}^{2}}{{\left(\omega_{j}^{\mathrm{SC}}\right)}^{2}}$$

where $\omega_{j}^{\mathrm{OC}}$ and $\omega_{j}^{\mathrm{SC}}$ are angular frequencies with open-circuit and short-circuit, respectively. We will reveal (1) how is this factor related to the maximum ability of external capacitance to change the natural frequencies and (2) how is this factor related to the geometric design of the piezoelectric materials.

Let us recall the dynamic equation of the piezoelectric structure with two electrodes (one voltage DOF):

(5) $$\begin{array}{c} M\ddot{x}+Kx-\eta V=f\left(t\right)\\ C_{p}V+\eta^{T}x=Q\left(t\right) \end{array}$$

where M and K are the mass and stiffness matrices, respectively; η is the piezoelectric matrix; x is the displacement vector; *V* is the voltage between the electrodes; f(t) is external force vector; Q(t) is the electric quantity of the circuit connected to the piezoelectric patch; and $C_{p}$ is the intrinsic capacitance. Shunting an external capacitance $C_{e}$ gives an additional equation:

(6) $$Q\left(t\right)=-C_{e}V$$

and thus Q(t) in the second equation of (5) can be eliminated, and the equation becomes

(7) $$\left(C_{p}+C_{e}\right)V+\eta^{T}x=0$$

If the external capacitance $C_{e}=+\infty$, the voltage between the piezoelectric electrodes is zero and this makes the piezoelectric patch short circuit. Accordingly, Equation (5) becomes

(8) $$M\ddot{x}+Kx=f\left(t\right)$$

Otherwise, if the external capacitance $C_{e}=0$, the piezoelectric patch is open circuit and Equation (5) becomes

(9) $$M\ddot{x}+\left(K+C_{p}^{-1}\eta^{T}\eta \right)x=f\left(t\right)$$

It is clear that when external $C_{e}$ varies in interval [0,+∞), it can only induce limited change to the stiffness and the natural frequencies can only vary in a limited zone. MEMCF is defined upon the maximum fraction of frequency changing.

Solving eigenvalue problem of Equations (8) and (9), respectively, we can get the angular frequencies $\omega_{j}^{\mathrm{OC}}$ and $\omega_{j}^{\mathrm{SC}}$. Owing to the orthogonality of the piezoelectric structure modal shape in open-circuit and short-circuit, we can get the following expression:

(10) $$\begin{array}{c} \phi_{\mathrm{OC},j}^{T}K\phi_{\mathrm{OC},j}+V_{j}C_{p}V_{j}=\omega_{oc,j}^{2}\phi_{\mathrm{OC},j}^{T}M\phi_{\mathrm{OC},j}\\ \phi_{\mathrm{SC},j}^{T}K\phi_{\mathrm{SC},j}=\omega_{\mathrm{SC},j}^{2}\phi_{\mathrm{SC},j}^{T}M\phi_{\mathrm{SC},j} \end{array}$$

where $\phi_{\mathrm{OC},j}$,$\phi_{\mathrm{SC},j}$ are the *j*th modal shapes with open circuit and close circuit respectively. We assume that the changing of electrodes status does not result in significant structuraldeformation difference, namely,


(11) $$\phi_{\mathrm{OC},j}\approx \phi_{\mathrm{SC},j}$$

This assumption is reasonable when the amount of piezoelectric materials are minor and it is the case in this paper.

Combining Equations (4) and (10), we can get


(12) $$\frac{V_{j}C_{p}V_{j}}{\phi_{\mathrm{SC},j}^{T}K\phi_{\mathrm{SC},j}}\approx \frac{{\left(\omega_{j}^{\mathrm{OC}}\right)}^{2}-{\left(\omega_{j}^{\mathrm{SC}}\right)}^{2}}{{\left(\omega_{j}^{\mathrm{SC}}\right)}^{2}}=k_{j}^{2}$$

This indicates that MEMCF $k_{j}^{2}$ also represents the proportion of the electric energy in the mechanical energy when the piezoelectric structure vibrates in the form of $\phi_{\mathrm{SC},j}$. In limited usage of the piezoelectric material, the geometry parameters of the piezoelectric materials, such as position and shape, do not change the mechanical energy of the structure significantly, but play a dominated role of the electric energy stored in the piezoelectric patches. The relation between the voltage, V, and the electric field intensity, E, is

**Figure 5:**
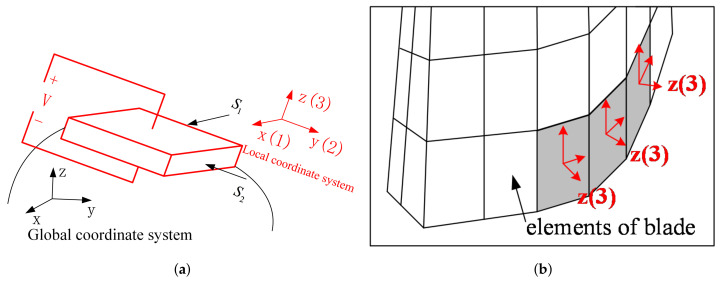
Illustration of the local coordinates on which the constitutive equation of piezoelectric materials is defined. (**a**) Local coordinates and the polarization direction. (**b**) Local coordinates on the blade.

(13) V=Ed

where d is the distance between the electrodes of the piezoelectric patch. Based on the constitutive relationship of the piezoelectric material, the electric field intensity, E, is the linear summation of the strain in every direction:

(14) $$E_{i}=\sum_{j=1}^{6}h_{\mathrm{ij}}S_{j}$$

where $h_{\mathrm{ij}}$ is piezoelectric constant and i refers to the direction of the electric field. The local coordinate system defined by the polarization direction of the piezoelectric material is shown in Figure 5a, the constitutive relationship of the piezoelectric material is expressed in the local coordinate system. For instance, when the polarization direction is z (3), the direction of the electric field is set up in the direction z (3), which means the electric field intensity E in Equation (13) is $E_{3}$.

Therefore, based on the strain distribution of the structure, we can determine the place to arrange the piezoelectric material. Namely, piezoelectric materials should be placed at the area with large $|E_{3}|$ of the blade to achieve the highest possible modal electromechanical coupling factor. In addition, we assume that the modal strain distribution would not be significantly altered by the introduction of piezoelectric materials. Therefore, $|E_{3}|$ can be estimated by the modal strain field of the structure before the installation of piezoelectric materials. In this way, the priority places for the installation of piezoelectric materials is determined by a single modal analysis and minor additional computing (Equation (14)). The detailed procedure will be enclosed in the next section.

### 3.2. The Optimization Procedure

The topology optimization for the piezoelectric material (PZT-5H is used, as shown in Appendix A) on the bladed disk can be done based on FEM model. The piezoelectric materials are introduced by replacing elements (if they are embedded) or adding an extra layer of elements (if they are bonded). In order to not weaken the strength of the blades or not add too much weight to the whole structure, we set the ratio, $R_{m}$, of the piezoelectric material mass on a single sector, $m_{\mathrm{pzt}}$, and the blade mass on a single sector (does not include disk), $m_{\mathrm{blade}}$, as our design constrain:

(15) $$R_{m}=\frac{m_{\mathrm{pzt}}}{m_{\mathrm{pzt}}+m_{\mathrm{blade}}}$$

When the blades vibrate, the induced strain mainly happens along the blade surface. In the normal direction of the blade, the strain is small. Therefore, the working mode of the piezoelectric elements is ‘3-1’ mode, so the electric field intensity E in Equation (12) is $E_{3}$. Moreover, the polarization direction of the piezoelectric elements is along the normal direction of the blade aera, and the normal direction is set to the outward of the blade surface (as shown in Figure 5b).

The objective function of the optimization is

(16) $$\mathrm{Obj}:\max(\sum_{\mathrm{elem}}|E_{3}|)$$

As discussed, this can maximize the MEMCF and endow the largest possible capability for the an external capacitance to tailor the natural frequencies of the structure.

**Figure 6:**
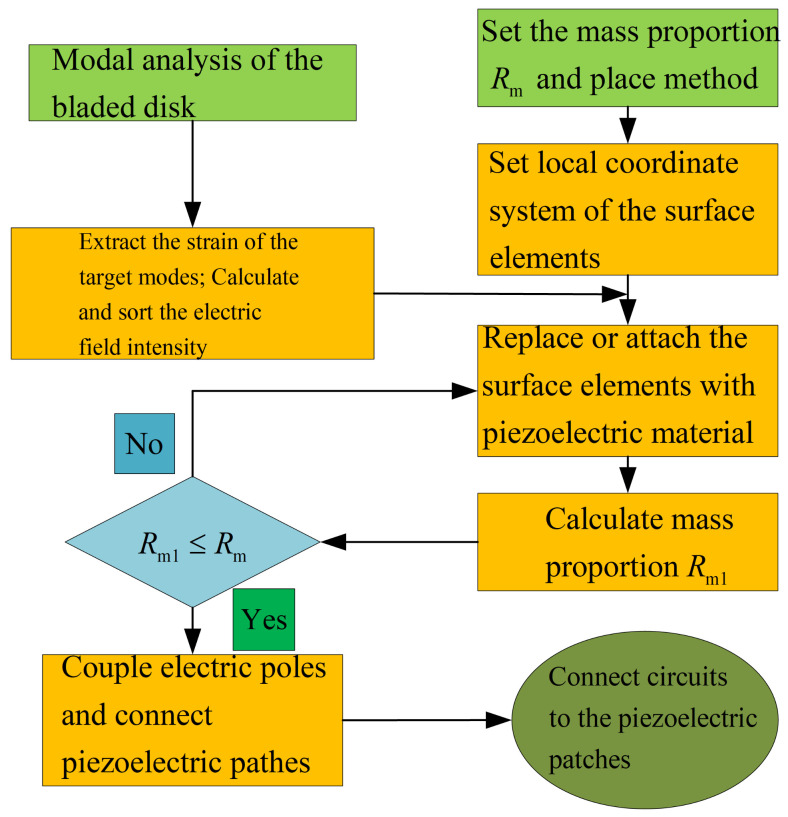
Optimization procedure of the piezoelectric materials distribution on the blades.

**Figure 7:**
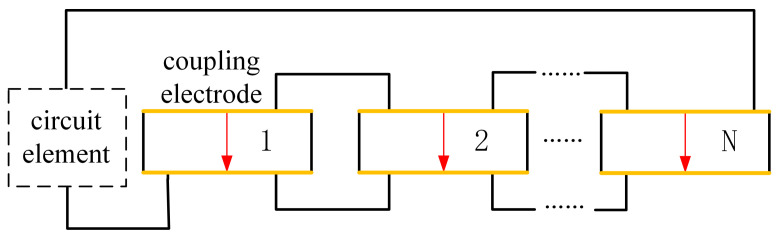
The electric connection of several distributed piezoelectric patches to the same circuits.

Based on the FEM model of the bladed disk, the position to place the piezoelectric elements is decided by the element strain. The optimization procedure is given as follows (shown in Figure 6):

1. Conduct modal analysis of the tuned bladed disk and obtain its modal information. This can be done with the sector model plus periodic conditions as shown in Figure 1b. Our target modes are those dominated by the blade 1st bending deformation (1st modal family).
2. Extract the blade surface elements strain. It should be noted that the strain need to be calculated at the element local coordinate system, and the way to set local coordinate system is shown in Figure 5b. Use the strain to calculate the electric field intensity $|E_{3}|$ of each element based on Equation (12). Then, sort the element according to the electric field intensity $|E_{3}|$. Note that element strain is an averaged value from the distributed strain in the element. In principle, it could even generate a null field (for example in a cantilever beam, if the element is located at the neutral axis in a pure bending). To avoid this abnormal situation, it is suggested to have a finer mesh so that the strain does not vary significantly inside a single element.
3. Choose the way to place the piezoelectric material.
  (a) If the piezoelectric materials are embedded to the blades. First, set the elements coordinate system of the blade surface elements. The z direction of the local coordinate system is along the outward normal direction of the blade surface. Then, replace the blade surface elements with the piezoelectric material elements. In ANSYS, this means changing element type from SOLID185 to SOLID226. The criterion is replacing the blade element based on the electric field intensity $|E_{3}|$ from the largest ones until the mass ratio $R_{m}$ meets the condition.
  (b) If the piezoelectric materials are bonded to the blades. We generate new elements along the norm direction of the blade elements. Namely, we will create a new layer of elements on the installation place. The remaining operations are the same as when we embed the piezoelectric materials.
4. Simulate the electrodes. The electrodes applied on a continuous area of piezoelectric materials are modeled by coupling the voltage DOFs on the top and bottom surfaces.
5. Interconnect discontinued piezoelectric materials on the blade. After the optimization, there may be several discontinued areas of piezoelectric materials. We will interconnect their electrodes so there is only one port on each blade, as shown in Figure 7. To do this, the poling direction of some areas should be reversed so that all the areas have the same signs of charges. Based on the sign of electric field intensity $E_{3}$, we can judge the poling direction.

### 3.3. Optimized Distributions of Piezoelectric Materials on the Blade

**Figure 8:**
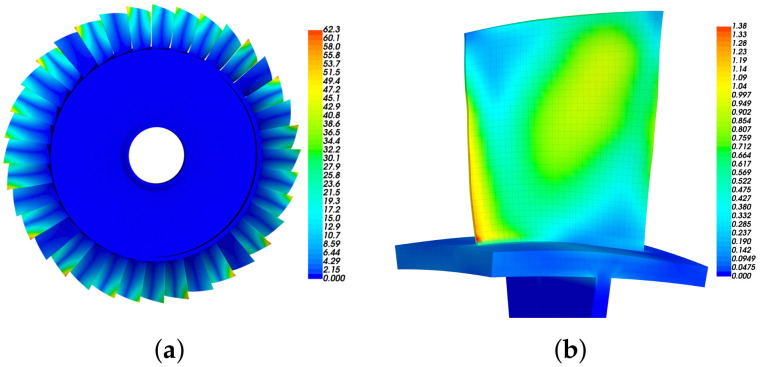
The modal deformation of the bladed disk dominated by the blade 1st torsional mode (marked with orange circle in Figure 2a). (**a**) Overall modal displacement. (**b**) Total strain on a single blade.

**Figure 9:**
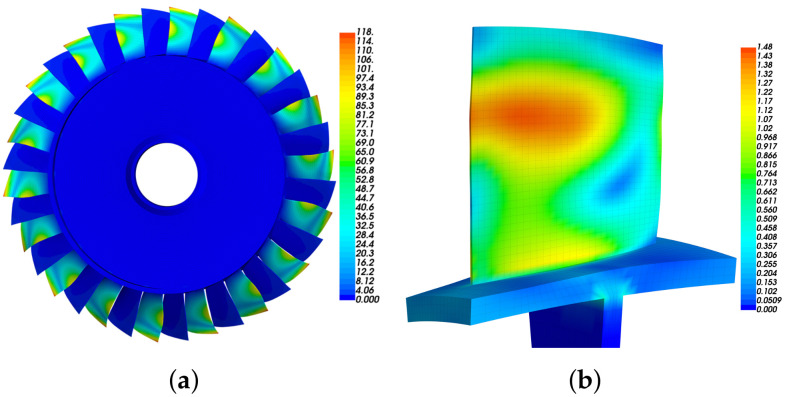
The modal deformation of the bladed disk dominated by the blade 2nd bending mode (marked with green circle in Figure 2a). (**a**) Overall modal displacement. (**b**) Total strain on a single blade.

Although our primary object is the first modal family (1st blade bending), the proposed algorithm can be used to any modes. To illustrate this, we optimize the distribution of piezoelectric materials for the first three modal families. The deformations shown in Figure 8a and Figure 9a are the modal shapes in the second and third modal families, respectively. We can see that the second modal family of the blade disk is dominated by the blade 1st torsional deformation, and the third modal family is dominated by the blade 2nd bending deformation.

**Figure 10:**
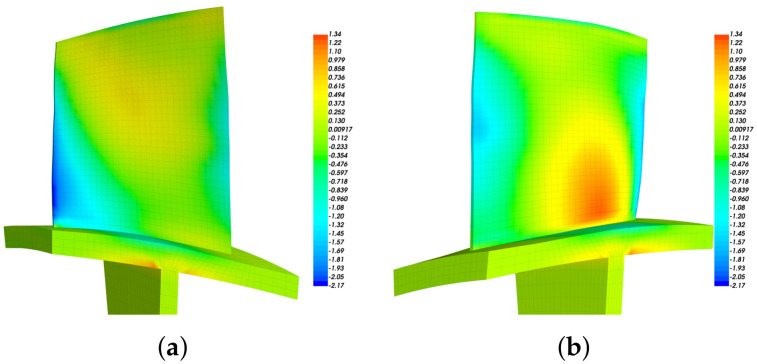
The electric intensity $E_{3}$ for the blade 1st torsional mode. (**a**) Pressure side. (**b**) Suction side.

**Figure 11:**
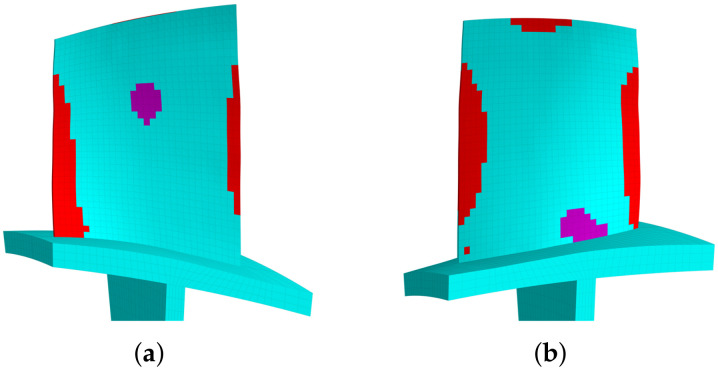
The optimized distribution of the embedded piezoelectric material for the 2nd modal family dominated by the blade 1st torsional mode (mass ratio = 10%). (**a**) Pressure side. (**b**) Suction side.

**Figure 12:**
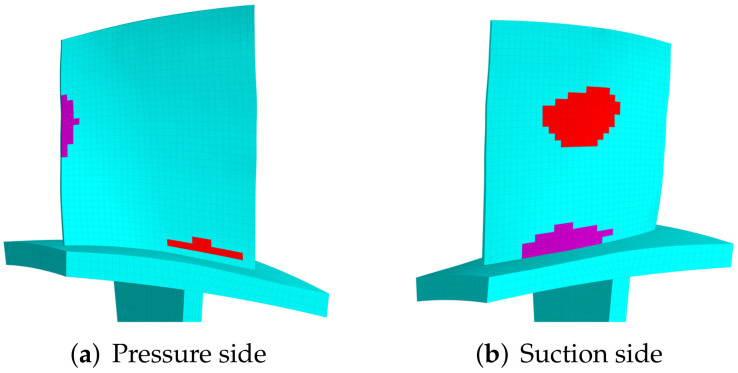
The optimized distribution of the embedded piezoelectric material for the 3rd modal family dominated by the blade 2nd bending mode (mass ratio = 10%). (**a**) Pressure side. (**b**) Suction side.

The modal strain distributions are also given in Figure 8b and Figure 9b. Note that the absolute values have no physical meaning, and we only use the relative values to determine the priority of locations to introduce piezoelectric materials. The priority indicator $E_{3}$ are computed according to Equation (15) and the results are shown in Figure 10. The optimized distributions of piezoelectric materials are shown in Figure 11 and Figure 12, where different colors (red and purple) indicate the piezoelectric materials with opposite polarization directions. The best locations results follow the distribution of large $E_{3}$ values as imposed by the algorithms. Thus, the agreements between Figure 10 and Figure 11 indicate that the algorithm is performed as expected. According to Equation (15), indicator $E_{3}$ can also be regarded as a weighted sum of the strain components. Thus, the distribution of $E_{3}$ should also be similar to (but not necessarily the same as) the distribution of strain. This remark can be verified by Figure 8b and Figure 10a.

**Figure 13:**
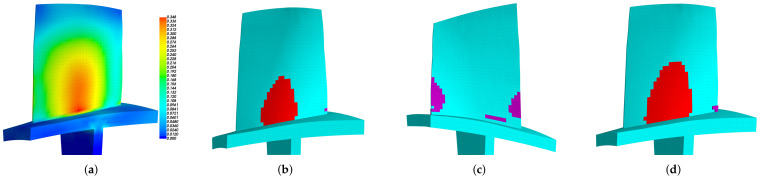
The optimized distribution of piezoelectric materials for the 1st modal family, mass ratio = 10%. (**a**) $E_{3}$, (**b**) Embedded Pzt (Suction side), (**c**) bonded Pzt (Pressure side), (**d**) bonded Pzt (Suction side).

Eventually we optimize the distribution of piezoelectric materials for the first modal family, which is chosen to illustrate the performance of the proposed method in the later sections. The results are shown in Figure 13b for the embedded case. We can see that the suggested area is not completely located at the root area of the blade, but is expands from the root to center of blade. Such results can be explained by $E_{3}$ shown in Figure 13a. The suggested distribution for the bonded case are given in Figure 13c,d. We can also see that the placement method would not significantly change the distribution area of the piezoelectric materials.

**Figure 14:**
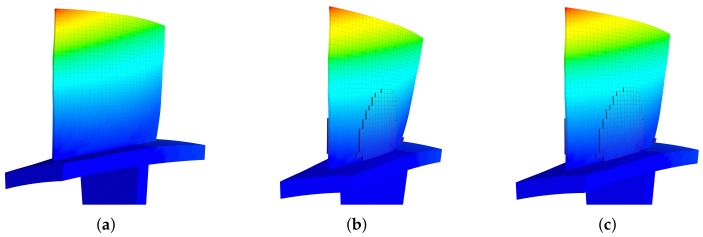
The modal shapes of the 1st modal family at different situations, mass ratio = 10%. (**a**) Original, (**b**) Bonded Pzt, open-circuit, (**c**) Bonded Pzt, short-circuit.

**Figure 15:**
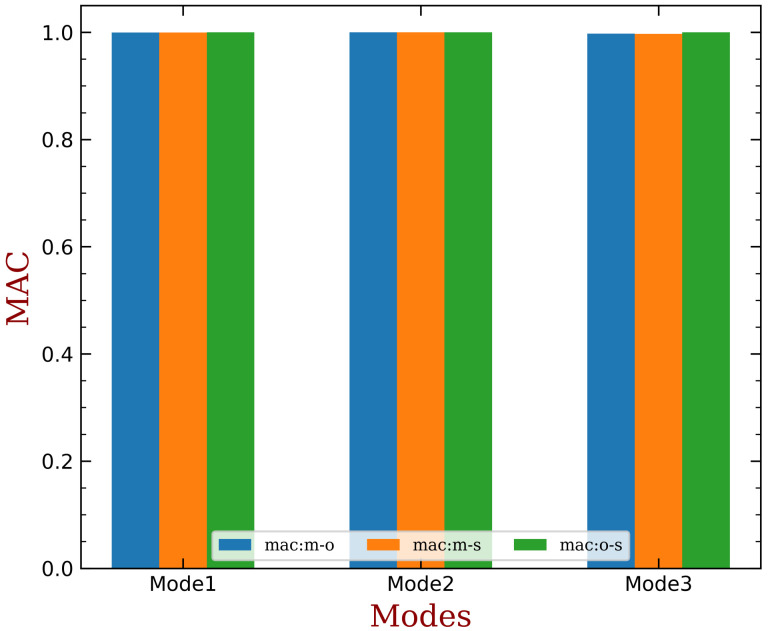
The modal assurance criterion (MAC) between the original (denoted by ‘m’), open-circuit (‘o’) and short-circuit (‘s’) modal shapes for the first three modal groups.

The modal shapes with piezoelectric materials being open-circuit and short-circuit are compared with the original blades in Figure 14. Little visual difference can be found among them, and we can use modal assurance criterion (MAC) to quantify their similarity:

(17) $$\mathrm{MAC}\left(a,b\right)=\frac{|a^{H}b|}{\left|a||b\right|}$$

where a and b are vectors whose similarity to be evaluated, superscript H refers to conjugate transpose. MAC varies from 0 to 1, and a closer value to 1 indicates a stronger similarity between a and b. Figure 15 summaries MAC values among the original, open-circuit and short-circuit modal shapes for the first three modal groups. We can see their MAC values are very close to 1. These results support the assumption that introduction of piezoelectric materials and the status of electrodes do not significantly change the modal shapes (when small amount of piezoelectric materials are used), as expressed in Equation (11).

**Figure 16:**
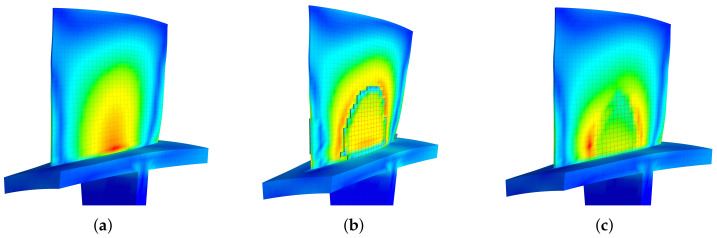
Total strain distribution of the 1st modal family at different situations, mass ratio = 10%. (**a**) Original, (**b**) Bonded Pzt, (**c**) Embedded Pzt.

Figure 16 demonstrates the strain distribution influence by the introduction of piezoelectric materials. The overall distributions of the train field are nearly the same when piezoelectric materials are introduced (bonding or embedding). Note that the edge of the piezoelectric area is determined by the edges of elements, so it is not smooth. Consequently, there will be stress concentration near the edges. This stress concentration only happens in the numerical simulation. In practical, one can choose to use the rounded or rectangular piezoelectric materials that can be purchased with ease, to cover the targeting area obtained by the proposed method. Alternatively, one can choose to customize the piezoelectric materials after smoothing the edges. On one hand, the similarity of the overall distribution before and after the introduction of piezoelectric materials is justifying the design procedure. On the other hand, it also explains why we use the modal strain field of the original blade to build our topological design. If we first introduce a small proportion of piezoelectric materials and the location of the remaining proportions are determined by the strain field of the blade with existing piezoelectric materials, such a ‘numerical’ stress concentration will mislead the design.

## 4. Intentional Capacitance Mistuning to Improve Aeroelastic Stability

### 4.1. The Relation between Frequency Deviation and Shunt Capacitance

The topology optimization of the piezoelectric materials on the blades allows us to change the natural frequency (or local stiffness) of blades to the maximum extent, under limited amount of piezoelectric materials. The modal frequency of each blade can be varied if we connect different capacitances to the piezoelectric materials on each blade. In this way, we can implement the desired mistuning pattern by capacitance mistuning. To do this, we need to quantify the relationship between the blade frequency with respect to the capacitance. Namely, for the desired mistuning pattern (normally given by the distribution of frequency deviation among sectors, $\Delta \omega_{j}$ with j=1,2,… the sector index), we can implement it by a distribution of capacitance among sectors ($C_{j}$ with *j* the sector index) but we must know the relationship between $C_{j}$ and $\Delta \omega_{j}$.

**Figure 17:**
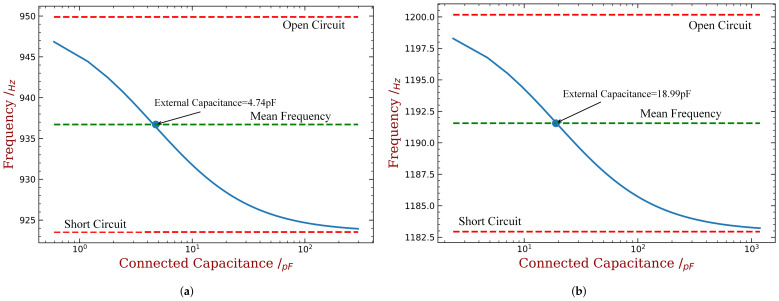
The relation between the blade 1st modal frequency and the shunt capacitance, mass ratio = 10%. (**a**) Embedded PZT and (**b**) bonded PZT.

The relation between the first modal frequency of the cantilever blade and the capacitance is shown in Figure 17a (embedded pzt) and Figure 17b (bonded pzt). The usage of the piezoelectric material are both 10%. Such results can be obtained by solving the eigenvalue problem associated with Equations (5) and (6) for each given capacitance $C_{e}$. The relation between the first modal frequency of the cantilever blade and the capacitance is shown in Figure 17a (embedded pzt) and Figure 17b (bonded pzt). The usage of the piezoelectric material are both 10%. Such results can be obtained by solving the eigenvalue problem associated with Equations (5) and (6) for each given capacitance $C_{e}$. The modal frequency of the cantilever blade can be changed from around 924 to 946 Hz by varying the external capacitance for the embedded case. Such a range may not be said wide in general situations, but it is acceptable to create an intentional mistuning. If not satisfied, one can increase this range by using more piezoelectric materials (increasing the value of $R_{m}$ in the topological optimization), or by using more powerful piezoelectric materials. More importantly, such a varying range of modal frequency is achieved by a much wider range of the capacitance. Please note that the logarithmic scale is used in Figure 17a,b. This means that the value of capacitance can vary within 3 orders of magnitude to achieve such a 20/950 difference in natural frequencies. Therefore, the design capacitance can subject to relatively large uncertainties and would not induce significant error to the desired mistuning pattern. This endows a much higher robustness of the proposed method over the mechanical manufacturing approaches. Similar remarks can be given to the bonded PZT case, where the frequency range is slightly narrower but the overall trend is the same.

In summary, we will set a mean frequency that all of the blades on the bladed disk should be first tuned with same external capacitance. In this way, we can additionally modify the connected capacitance to realize the desired intentional mistuning. The capacitance with 4.74 pF and 18.99 pF are chosen as the mean values for the embedded and bonded cases respectively, as shown in Figure 17.

### 4.2. Mistuning Pattern Design

We follow the literature [14,15] and employ mistuning patterns with harmonic (sinusoidal) forms:

(18) $$\Delta \omega_{j}=A*\sin\left(\frac{N}{2\pi }hj+\theta \right)$$

where N is the overall number of blades and equals to 36 in this paper; j=1,2,…N is the sector index; h is the harmonic index and represents the repeating times of pattern along the circumference direction; θ is an arbitrary starting phase and it does not affect the results; A is the amplitude of mistuning. After obtaining the knowledge about modifying the blade frequency with external capacitance, we can implement such an intentional mistuning pattern. As we assume to fully use the adjust range of frequency to maximize the strength of mistuning, A is determined by Figure 17. Then, what remains is only to determine harmonic index h.

**Figure 18:**
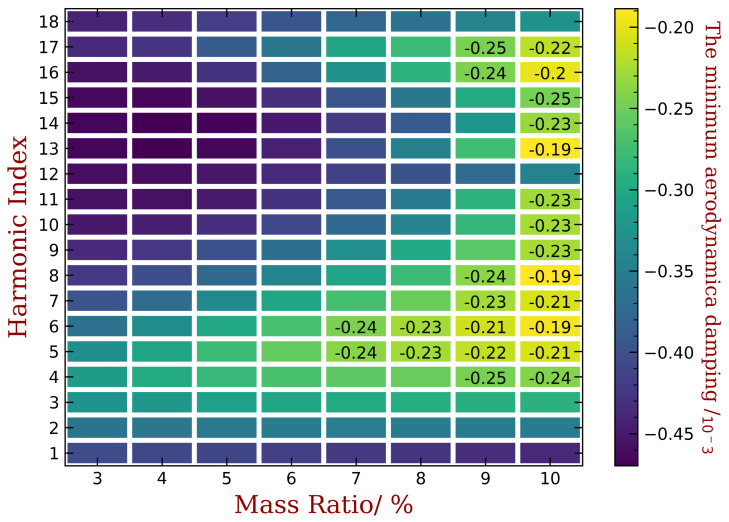
The variation of $\xi_{\min}$ with respect to harmonic index h and mass ratios when piezoelectric materials are embedded into the blades.

**Figure 19:**
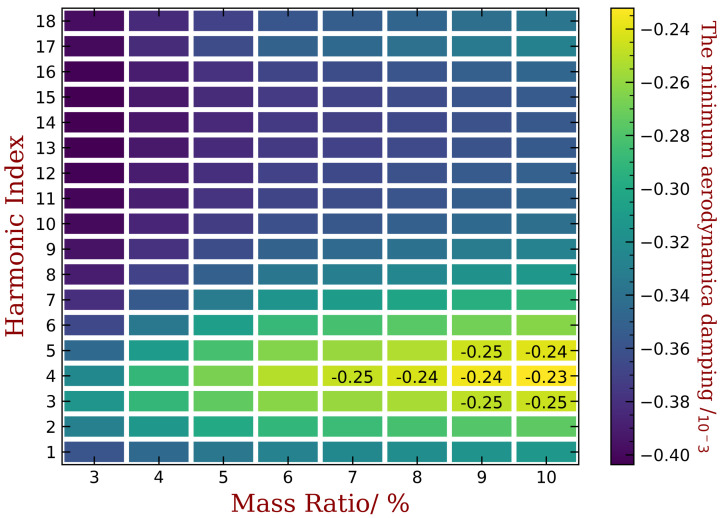
The variation of $\xi_{\min}$ with respect to harmonic index h and mass ratios when piezoelectric materials are bonded to the blades.

The aeroelastic stability of the bladed disk is determined by the minimum aerodynamic damping ratio $\xi_{\min}$. However, there is no general conclusion about the relationship between $\xi_{\min}$ and harmonic index h of mistuning. Therefore, we conduct parametric studies to determine the best choice of h for the largest possible $\xi_{\min}$. In our investigation, we also varied the mass ratios of the piezoelectric material from 3% to 10%. The results are summarized in Figure 18 for embedded PZT and Figure 19 for bonded PZT. In each figures, the x-coordinate refers to different mass ratio, and the y-coordinate is the harmonic index for which the capacitance varies along the circumference direction. The color is indicating the magnitude of $\xi_{\min}$ when (1) the piezoelectric materials are optimized under the mass ratio shown in the corresponding x-coordinate, and (2) the mistuning pattern is following the harmonic index shown in the corresponding y-coordinate. We have also labeled the values when $\xi_{\min}$ is greater than −0.25‰, while the values of unlabeled colors can be estimated by the scale shown alongside the figure.

In both figures, the best results of $\xi_{\min}$ are still negative values, this means that the system is still unstable. Note that the original $\xi_{\min}$ for the tuned bladed disk is lower than −0.5‰, as shown in Figure 4b. Thus, a $\xi_{\min}$ in the mistuned cases larger than −0.5‰ can be regarded as an improvement of the aeroelastic stability. The remaining negative aeroelastic damping is easier to compensate by material or structural damping. Moreover, we can increase the mass ratio or use more powerful piezoelectric materials. In this paper, we just use NASA-rotor37 blades as example and choose an abnormal working condition to reproduce the flutter and to illustrate the performance of the proposed method. Therefore, the observation of the improved $\xi_{\min}$ in such a unstable situation is sufficient here.

When the mass ratio is low, for example, when it equals to 3% and 4%, the improvement of $\xi_{\min}$ is not significant. Despite that, the performance of some harmonic indexes is better than others, for example when it equals to 3, 4, 5 or 6. These is consistent with the engine order of the modes with higher aeroelastic damping in the tuned case as shown in Figure 4b. Such an consistency is the same with previous studies concerning harmonic mistuning [14,15,33]. Under each level of mass ratio, the distribution of piezoelectric materials are optimized using the aforementioned approach. Therefore, a larger mass ratio means a greater gap between the OC and SC natural frequencies and mistuning amplitude A in Equation (17) is lager. Consequently, we can observe in both figures that the best $\xi_{\min}$ increase monotonously with the increase of mass ratio of piezoelectric materials. For example, in Figure 18, $\xi_{\min}$ equals to −0.24‰, −0.23‰, −0.21‰ and −0.19‰ when mass ratio $R_{m}$ equals to 7%, 8%, 9% and 10%, respectively. In addition, the best choice of the harmonic index h does not vary significantly for different mass ratio, and the performance difference among harmonic indexes is less apparent when mass ratio is increasing. This trend is less significant for the bonded PZT case (Figure 17) because it provides weaker frequency gap than the embedded case under the same mass ratio.

**Figure 20:**
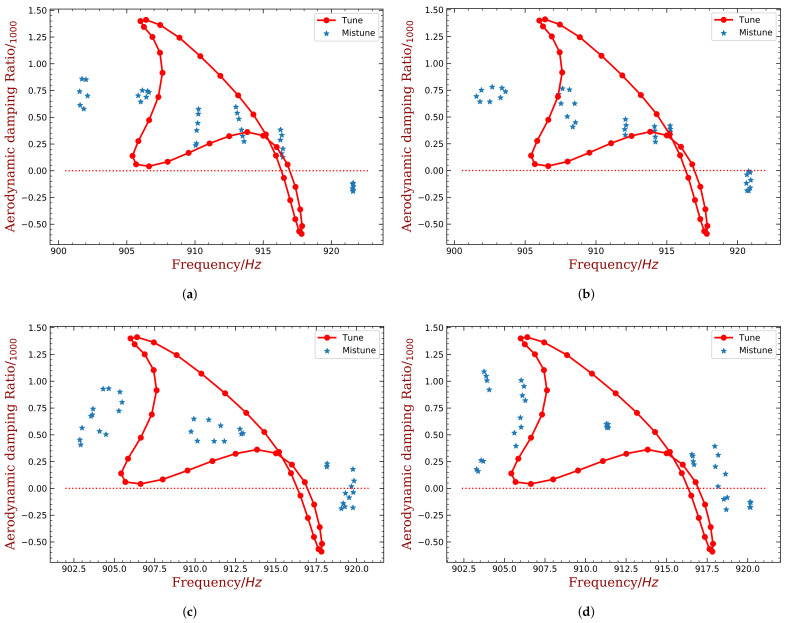
The distribution of aerodynamic damping ratio with different harmonic index. (mass ratio = 10%, embedded PZT). (**a**) h=6, (**b**) h=8, (**c**) h=13, (**d**) h=16.

**Figure 21:**
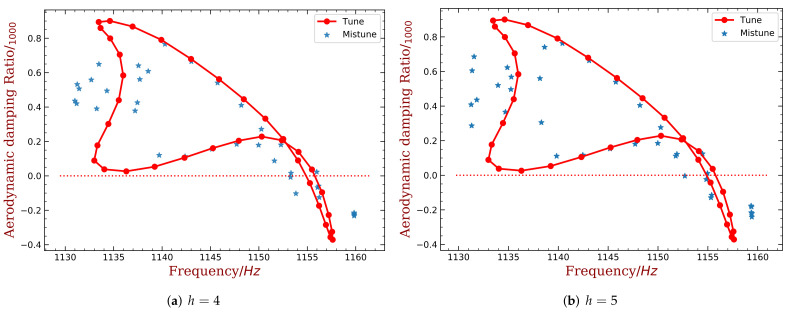
The distribution of aerodynamic damping ratio with different harmonic index. (mass ratio = 10%, embedded PZT). (**a**) h=4, (**b**) h=5.

**Figure 22:**
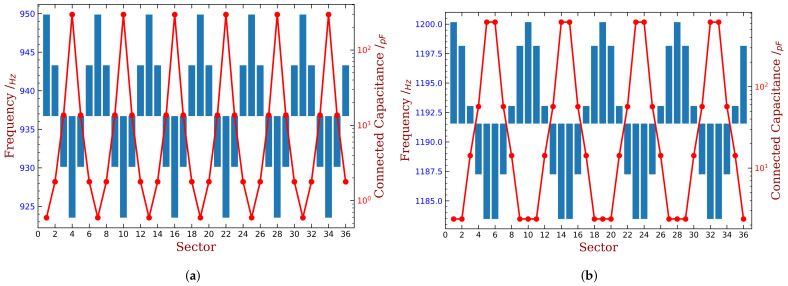
Illustration of some intentional mistuning patterns (blue bars) and associated implementation by capacitance (red dots), mass ratio = 10%. (**a**) h=6, embedded PZT, (**b**) h=4, bonded PZT.

Finally, the desired harmonic indexes are 6, 8, 13 and 16 for 10% mass ratio, when the piezoelectric material is embedded. Compared with the original results shown in Figure 4b, the amplitude of $\xi_{\min}$ has been reduced by half. When the piezoelectric material is bonded, the best harmonic index are 4 and 5. Detailed results are shown in Figure 20 and Figure 21. We can see that these intentional mistuning can increase the minimum aerodynamic damping to higher levels compared with the tuned bladed disk. Moreover, the number of the unstable mode decreases after introducing the intentional mistuning. With h decided, the mistuning patterns can be implemented, as illustrated in Figure 22. Recall that the capacitance is computed by the blade frequency variation according to Figure 17. Please note that the required capacitance is varying from several to hundreds pF, and this can be purchased with easy.

**Figure 23:**
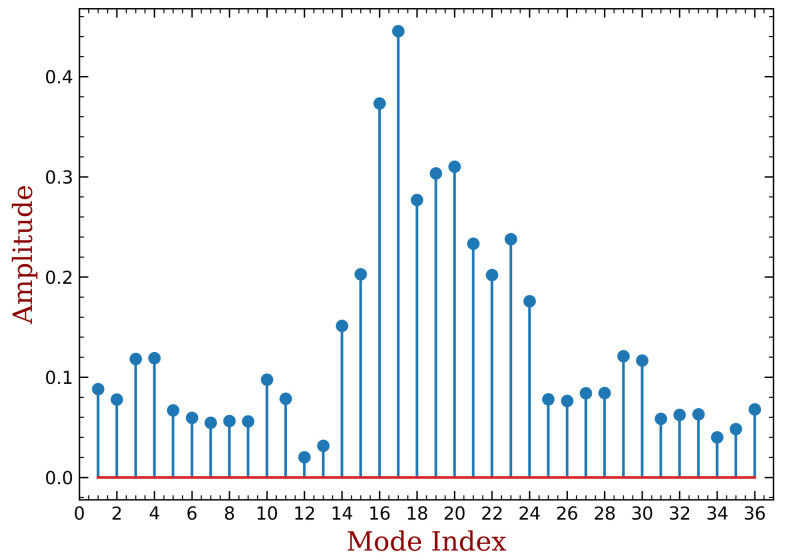
The contributions of tuned modes to the minimum aerodynamic damping (mass ratio = 10%, h=13, bonded PZT).

We can decompose the mistuned modal shape corresponding to $\xi_{\min}$ by the tuned modal shapes, as shown in Figure 23. We can see that a lot of modes are involved including those with positive aeroelastics damping ratios. With their contributions, the stability of mistuned modes are improved. This is also consistent with the existing literature.

### 4.3. Effects of Random Mechanical Mistuning

As mentioned, mechanical mistuning caused by the material disperse, wear and manufacturing tolerance is random and inevitable. Therefore, the performance of intentional mistuning should be further examined in the presence of random mechanical mistuning. We apply the optimum intentional mistuning to the bladed disk obtained in the last section and introduce random mechanical mistuning simultaneously. The former is realized by external capacitance and the latter is simulated by stiffness variance in the numerical analysis. The level of random mistuning is quantified by the standard deviation of the first modal frequency with cantilevered blade along sectors. There are many sources of mistuning in practice as mentioned. Quantifying each of them in real engineering scenario can be a difficult task, and it is out of the scope of this work. However, their influences can be eventually attributed to the non-periodic perturbation on the stiffness and inertial coefficients of the dynamic model. Therefore, we use stiffness mistuning as a representative case to study the influence of additional random mistuning caused by different sources. Such an idea can be seen in various published papers [37,38] particularly when the researchers aim to draw general conclusions concerning the influence of mistuning with respect to the dynamic characteristics.

**Figure 24:**
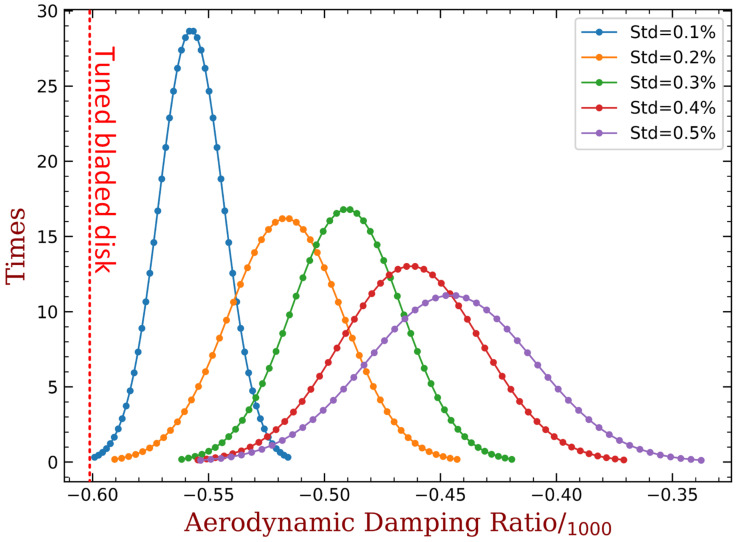
The frequency graph of $\xi_{\min}$ only with mechanical mistuning at different level.

At first, we only introduce mechanical mistuning into the tuned bladed disk. We take 100 groups of $\Delta \omega_{j}$, j=1,2,…,36 from the normal distribution with 0 mean expectation at each standard deviation changes from 0.1% to 0.5%. Note that when the standard deviation equals to 0.5%, most of the $\Delta \omega_{j}$ values will be located approximately in the same range as that can be expanded by the capacitance as shown in Figure 17. That is why we choose to analyze this range of standard deviation. The frequency graph is shown in Figure 24, where the original $\xi_{\min}$ value of the tuned bladed disk is also marked. The y-axis label ‘times’ in these figures refers to the count of the samples whose $\xi_{\min}$ is inside the corresponding small interval between two markers in the curves. Thus, the results shown in these figures can be interpolated as an approximation of the probability density function. We can see that mistuning is always beneficial to the improvement of aeroelastic stability in comparison with the tuned case, and this observation is consistent with the literature [1,2]. Specifically, the mean value of $\xi_{\min}$ in creases with respect to the mistuning level and the samples are distributed in a wider range. As mentioned, the variance range of blade frequencies with 0.5% random mistuning is equivalent to it shown in Figure 24. But 0.5% random mistuning can only have mean value of $\xi_{\min}$ around −0.44‰ and barely reaches −0.35‰. This is much weaker than the performance of intentional mistuning with harmonic form, where $\xi_{\min}$ can be increased to around −0.20‰ as shown in Figure 18 and Figure 19. Such a comparison also indicate the advantage of intentional mistuning.

**Figure 25:**
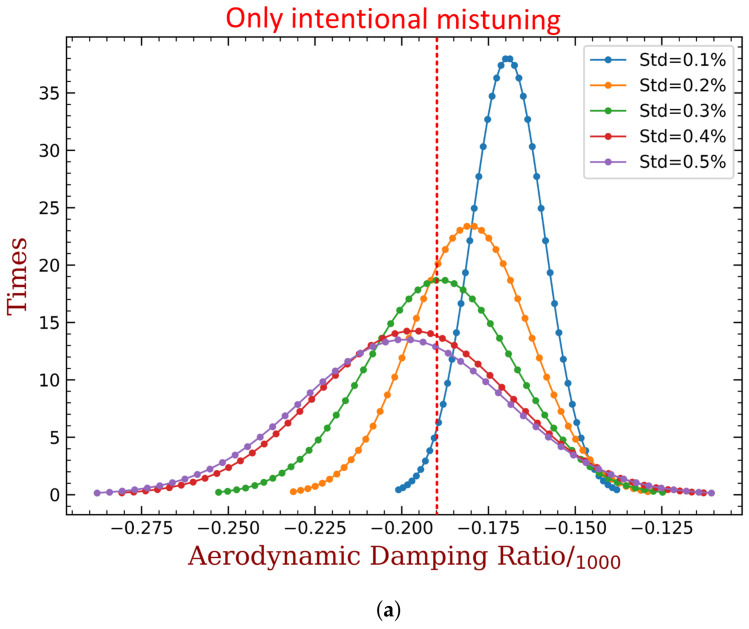

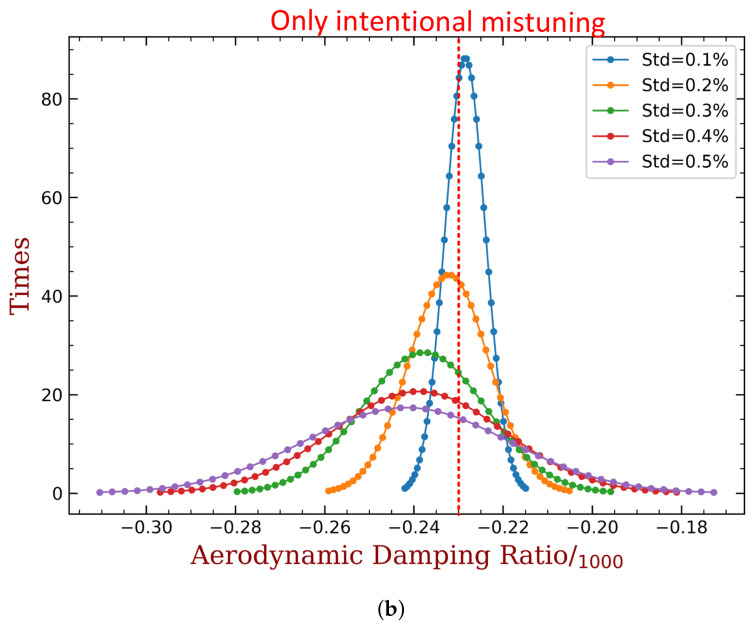
The frequency graph of $\xi_{\min}$ with best intentional mistuning (mass ratio = 10%) plus different level of mechanical mistuning. (**a**) h=6, embedded PZT; (**b**) h=4, bonded PZT.

Then we investigate the influence of mechanical mistuning when the intentional mistuning (implemented by capacitance) has already been imposed. The mass ratio of piezoelectric materials are both 10%, and we choose two typical cases from Figure 18 and Figure 19 as the intentional mistuning: (1) h=6 with embedded PZT, and (2) h=4 with bonded PZT. The results are shown in Figure 25, where the $\xi_{\min}$ with only intentional mistuning are marked as reference. When the random mistuning is small, the influence is generally small. Especially in the case of embedded PZT, most of the samples are better than the reference. When the mistuning level increases, the results start to spread, and nearly half of samples have worse performance than the reference. Despite that the $\xi_{\min}$ decreases from −0.23‰ to around −0.30‰, and the worst case is still higher than the best case in Figure 25. This indicates that intentional mistuning can still significantly improve the aeroelastic stability with the presence of random mechanical mistuning.

## 5. Conclusions

1. Herein, an adaptive method based on the piezoelectric technique to improve the aeroelastic stability of the bladed disk is proposed. The basic idea is to bond or embed piezoelectric materials to each blade and use different shunt capacitance on each blade as the source of mistuning. We show that the required small difference of stiffness among blades is altered into a relatively larger difference of the shunt capacitance. This provides a more feasible and robust way to implement the intentional mistuning.
2. A method to determine the distribution of piezoelectric materials have been established. A linearly weight strain indicator is used as the optimization criterion, whose physical meaning is the absolute value of the electric field. The theoretical basis of this method is clarified. The method is adapted for FE model and has no assumptions on the geometrics of piezoelectric materials. It only needs a single modal analysis of the bladed disk and a given threshold of the mass ratio, and eventually yields the best distribution of piezoelectric materials for the targeting mode(s). The obtained shapes are described by the edges of the finite elements so they are non-smooth. One can choose to use the rounded or rectangular piezoelectric materials that can be purchased with ease, to cover the targeting area obtained by the proposed method. Alternatively, one can choose to customize the piezoelectric materials after smoothing the edges. In either way, the proposed method can provide a good starting point.
3. An empirical bladed disk with NASA-ROTOR37 profile is used as an example. For the first bending mode of the blade, only using piezoelectric materials with a mass of 10% of the blade mass can reduces the unstable margin from 0.6‰ to 0.2‰. The required capacitance is varying from several to hundreds pF, and this can be purchased with easy. These quantitative results demonstrate that the proposed method is very promising.
4. The proposed method shows good robustness with the presence of random mechanical mistuning. We show that additional (and inevitable) mistuning would somewhat weaken the performance of intentional mistuning imposed by capacitance variance. However, the stability of the system is still much better than the original case.
5. There are two main challenges to be resolved before the proposed method can be further applied to real engineering products. The first one is the installation of piezoelectric materials (PZT, MFC and so on). In the laboratory environment, they are often glued to the surface of host structures, and additional protection is required when testing in rotation status [39]. Alternatively, the piezoelectric materials can be embedded into composites [40], and thus avoid negative affects to fluid field. In this way, the wire and electric components can also be packaged. The reliability of such installation strategies needs thorough validation before they can be used in real aero-engines. The second challenge is the strength of piezoelectric materials. We should locate them to the places where stress level is relatively high, to achieve a stronger ability of the electric field to tailor the mechanical properties. In this case, whether the piezoelectric materials and the glue can withstand the tress level for a satisfying duration should be investigated. We are addressing this issue with on-going experiments.

## Data Availability

The data presented in this study are available on request from the corresponding author..

## Appendix A. Material Properties of PZT-5H

Mass density: ρ=7500 kg/m^3^.

Material stiffness matrix evaluated at constant electric field:

$$c^{E}=10^{10}\times \left[\begin{array}{cccccc} 12.6 & 7.95 & 8.41 & & & \\ 7.95 & 12.6 & 8.41 & & & \\ 8.41 & 8.41 & 11.7 & & & \\ & & & 2.3 & & \\ & & & & 2.3 & \\ & & & & & 2.35 \end{array}\right]\mathrm{Pa}$$

Permittivity matrix evaluated at constant strain:

$$\varepsilon^{S}=\varepsilon_{0}\times \left[\begin{array}{ccc} 1700 & & \\ & 1700 & \\ & & 1470 \end{array}\right]$$

where $\varepsilon_{0}=8.854\times 10^{-12}$ C/(V·m).

Piezoelectric stress coupling matrix:

$$e=\left[\begin{array}{ccc} & & -6.5\\ & & -6.5\\ & & 23.3\\ & 0 & \\ & 17 & \\ 17 & & \end{array}\right]N/\left(V\cdot m\right)$$
